# Artificial intelligence, robotics, and person-centered care in nursing and midwifery education: qualitative study to develop an augmented caring pedagogy model

**DOI:** 10.1186/s12909-026-08717-7

**Published:** 2026-02-09

**Authors:** Tuba Sengul, Betül Uncu, Seda Sarıköse, Nurten Kaya, Violeta Lopez, Holly Kirkland-Kyhn

**Affiliations:** 1 Department of Nursing, Koç University School of Nursing, Topkapı, Davutpaşa Street. No:4, Istanbul, 34010 Türkiye; 2 Faculty of Health Science, Department of Midwifery, Istanbul University-Cerrahpasa, Istanbul, Türkiye; 3 School of Nursing, Midwifery & Social Sciences, International Institute of Health Sciences, Rockhampton, Sri Lanka; 4 School of Nursing and Allied Medical Sciences, Holy Angel University, Angeles, Philippines; 5 The Betty Irene Moore School of Nursing at UC Davis, Sacramento, USA

**Keywords:** Artificial intelligence, Robotics-Supported care, Person-Centred care, Nursing education, Midwifery education

## Abstract

**Background:**

The rapid growth of AI and robotics is reshaping nursing and midwifery education, offering personalised and data-informed learning while raising ethical and relational challenges. These changes require educators to reassess their readiness and teaching approaches, highlighting the need to understand how faculty integrate AI into person-centred care. This study aimed to explore how nursing and midwifery educators perceive the role of artificial intelligence and robotics in person-centred care education and to generate empirical insights to inform the development of the Augmented Caring Pedagogy Model (ACPM).

**Methods:**

This qualitative descriptive study was guided by the Normalization Process Theory. Four online semi-structured focus groups were conducted with 20 nursing and midwifery academics recruited from nine universities across Türkiye. Purposive sampling was used, and the data were analyzed using an integrated inductive–deductive thematic approach supported by MAXQDA 24.

**Results:**

Four themes were identified: (1) Coherence: Making Sense of AI-and Robotics-Supported Person-Centred Care, (2) Cognitive Participation: Engaging with Technological Transformation, (3) Collective Action: Operationalizing and Sustaining AI Integration, and (4) Reflexive Monitoring: Learning Outcomes, Equity, and Ethical Responsibility in AI-Enhanced Education. Educators viewed artificial intelligence and robotics as enhancing learning and professional growth, while also raising concerns related to empathy, ethics, inequality, and technological dependence. These findings collectively informed the development of the ACPM.

**Conclusion:**

Integrating AI and robotics into nursing and midwifery education offers substantial potential to advance person-centred, evidence-based teaching, but it also introduces ethical, emotional, and structural complexities. The ACPM offers a novel pedagogical framework centred on human–technology synergy and a reflective adaptation cycle to support ethically grounded and sustainable integration of emerging technologies. Findings should be interpreted in light of the single-country context and the early stage of AI and robotics integration within educational practice.

**Supplementary Information:**

The online version contains supplementary material available at 10.1186/s12909-026-08717-7.

## Background

The rapid development of artificial intelligence (AI) and robotics technologies in healthcare is reshaping nursing and midwifery fields, as well as higher education programs in these disciplines [1]. While decision support systems, automation, and data analytics are transforming care processes in clinical settings [2], the impact of a similar transformation on teaching and learning processes in higher education has become a significant area of debate [3]. Person-centered care is an approach that prioritizes the individual’s values, preferences, autonomy, and holistic needs, and the reconciliation of the use of technology in education with this human dimension is a fundamental area of inquiry for faculty members [4, 5]. In the context of higher education pedagogy, AI has the potential to transform instructional design, assessment approaches, student autonomy, and critical thinking skills. Adaptive learning, simulation-based skills training, personalized feedback, generative AI-based language models, and virtual clinical scenarios are reshaping learning experiences and faculty roles [6, 7]. However, ethical and pedagogical risks, such as algorithmic bias, privacy, academic integrity, content accuracy, and student over-reliance on AI, require careful, ethically informed integration into education [8, 9]. Accordingly, the literature emphasizes that effective implementation depends on both pedagogical adaptation (e.g., critical digital literacy, ethical awareness, and redesigned learning processes) and faculty members’ technological readiness and pedagogical capacity [10–12]. Although the literature on AI and robotics in nursing and midwifery education is largely grounded in high-income Western settings [2, 3], emerging evidence from the Middle East and other low-income regions indicates comparable barriers, such as infrastructural variability, ethical uncertainty, and uneven preparedness among faculty and institutions for digital health education [12–14]. These studies show that AI in health professions education is shaped not only by technological availability but also by sociocultural, economic, and regulatory contexts, underscoring the need for context-sensitive pedagogical inquiry. Despite growing clinical application studies involving technological tools in nursing and midwifery, evidence on faculty members’ perceptions and experiences of pedagogical AI/robotics use remains limited [1, 15]. As a result, much of the literature emphasizes technical integration and skills development, while pedagogical models that reconcile technology with humanistic and ethical care are still underexplored [6, 11, 16]. In particular, how faculty members interpret, adopt, and incorporate these technologies into educational practices under conditions of uncertainty remains unclear [17, 18]. This gap highlights the need for theoretically informed pedagogical frameworks that move beyond technical integration to foreground ethical reflection, relational care, and professional identity formation within technology-enhanced education. To address this need, we use the Normalization Process Theory (NPT) developed by Murray et al. [19] to examine how AI and robotic systems become embedded in nursing and midwifery education. NPT is well-suited to emerging, complex innovations because it attends to how new practices are collectively understood, enacted, sustained, and evaluated in real-world institutional contexts [19]. Accordingly, NPT guides analysis of (a) coherence (how faculty understand AI/robotics), (b) cognitive participation (motivations to engage), (c) collective action (pedagogical and institutional work), and (d) reflexive monitoring (how effects are appraised), while capturing the sense-making and ethical negotiation required when technologies challenge established pedagogical norms and person-centered care values.

## Methods

### Aims

The aim of this study was to explore how nursing and midwifery faculty members make sense of the integration of AI and robotic technologies into person-centred fundamental care within educational contexts, using NPT as an analytic lens. Specifically, the study aimed to investigate participants’ perceived future preparedness regarding curriculum design, assessment, and clinical integration, as well as the barriers and facilitators shaping this process. Informed by these findings, the study also aimed to generate the conceptual foundations underpinning an Augmented Caring Pedagogy Model (ACPM) to support pedagogical innovation in nursing and midwifery education.

### Research questions

These research questions were structured according to the four core constructs of NPT coherence, cognitive participation, collective action, and reflexive monitoring, which together provide a comprehensive framework for understanding how complex innovations, such as AI- and robotics-supported person-centred care pedagogies, are implemented, embedded, and sustained within educational practice. Consistent with NPT, perceived barriers and facilitators were examined as integral elements shaping these processes, rather than as a separate analytical category.

*Guided by this framework*,* the study was organized around the following research questions*:

1. Coherence (Sense-making): How do nursing and midwifery faculty members make sense of the integration of AI and robotic technologies into person-centred fundamental care within the educational context?
2. Cognitive Participation (Engagement and Ownership): How do faculty members demonstrate engagement, ownership, and motivation toward incorporating these technologies into educational programmes?
3. Collective Action (Implementation): How do faculty members describe the practical work involved in implementing AI and robotic technologies in curriculum design, assessment processes, and clinical integration?
4. Reflexive Monitoring (Evaluation and Improvement): How do faculty members evaluate, reflect on, and adapt the use of these technologies in education and clinical practice over time?

### Study design

This study employed a qualitative descriptive design using semi-structured focus group discussions (FGDs) [20]. The constructivist paradigm informed the research approach, assuming that knowledge is co-constructed through participants’ shared experiences and the researcher’s interpretive engagement [21]. NPT [19] provided the analytical framework for the study. NPT informed the development of the interview guide, the organisation of the coding structure, and the interpretation of how faculty members made sense of, engaged with, operationalised, and evaluated AI- and robotics-supported person-centred care in educational contexts. NPT was also suitable for analysing conceptual, forward-looking, or rhetorical reflections, as the theory examines how individuals make sense of and engage with emerging practices even before full implementation. The study followed the Consolidated Criteria for Reporting Qualitative Research (COREQ) checklist [22] to ensure methodological rigor and transparency (Supplementary File 1).

### Sampling and recruitment

The study population comprised academics employed in nursing and midwifery faculties across Türkiye, from research assistants to full professors. We used purposeful criterion sampling [23] to recruit full-time faculty with ≥ 1 year of experience in nursing or midwifery education who had experience, knowledge, or engagement with AI/robotics integration in person-centred fundamental care or educational practices. Hands-on use of AI/robotics was not required; participants were eligible if they had engaged with the topic at conceptual, curricular, or organizational levels. Faculty not currently involved in teaching or curriculum-related roles (e.g., retired, administrative-only, or adjunct staff) and those with < 1 year of professional experience were excluded. To capture diverse perspectives, we sought variation in age, gender, academic rank, years of experience, and institutional type (public/private), reflecting differences in organizational culture and technological readiness. Consistent with the NPT orientation toward collective sense-making and engagement, we conducted focus group interviews to elicit shared reflections and group-level dynamics. Sample size was guided by information richness and thematic saturation, resulting in four focus groups comprising 20 faculty members. Theoretical saturation was defined as the point at which no new codes, themes, or conceptual insights emerged. After the third focus group, discussions largely repeated previously identified themes; the fourth focus group was used to confirm and consolidate the emerging thematic structure. Eligible faculty were identified via institutional websites and researchers’ professional networks and were invited by email with details on the study aims, voluntary participation, and confidentiality procedures. Snowball sampling was used to broaden access to faculty familiar with AI/robotics integration; referrals were cross-checked, and sample diversity was monitored to minimize clustering and reduce referral-related bias.

### Data collection

Data collection consisted of two distinct phases: a pilot phase conducted for instrument refinement and the main study phase. A pilot study was conducted with five faculty members (three from the Department of Nursing and two from the Department of Midwifery) to assess the clarity, relevance, and flow of the semi-structured FGD guide. Pilot participants were recruited independently of the main study sample and were not selected to represent institutional diversity. Based on their feedback, minor revisions were made to the wording and sequencing of questions to improve clarity and comprehensibility. Data generated during the pilot phase were used solely for refining the interview guide and were excluded from the final analysis. Following ethical approval, the main study data were collected between October 20 and 30, 2025, through four semi-structured FGDs, each comprising five faculty members (*n* = 20). Participants in the main study were drawn from 9 different nursing and midwifery faculties across Türkiye, ensuring variation in institutional type and educational context. The overall research process, including transcription, preliminary analysis, and manuscript preparation, was completed by December 10, 2025. All FGDs were conducted online via Zoom or Microsoft Teams to facilitate participation from diverse geographic regions. To support confidentiality and minimize the risk of institutional identification, participants joined the sessions using self-selected pseudonyms, and no institutional affiliations were disclosed or discussed during the focus groups. Video use was optional, allowing participants to choose whether to keep their cameras on or off throughout the sessions. Only audio recordings were made. Descriptive background information (e.g., academic role, years of experience) was collected through a brief form completed during an initial individual contact prior to the FGDs. In addition to qualitative data, this brief form included a short descriptive questionnaire to capture participants’ demographic and professional characteristics, as well as selected contextual indicators of AI and robotics integration across institutions. The questionnaire included single-item, self-reported 5-point Likert-type rating scales assessing perceived adequacy of technical infrastructure and institutional support (1 = very inadequate, 5 = very adequate). These quantitative descriptors were collected solely for descriptive and contextual purposes, were not subjected to inferential statistical analysis, and did not inform the qualitative coding or theme development. Rather, they were used to provide background context for interpreting participants’ qualitative accounts. This information was not shared in group discussions; therefore, participants were unaware of each other’s organizational backgrounds. The FGDs were facilitated by the SS and BU authors, one holding a Doctor of Philosophy (PhD) in Nursing and the other a PhD in Midwifery. Both facilitators are faculty members with formal training and prior experience in qualitative research and focus group moderation. Discussions followed a semi-structured guide aligned with the study aims and the four NPT constructs (coherence, cognitive participation, collective action, and reflexive monitoring). (Table 1. Semi-Structured Interview Guide on AI- and Robotics-Supported Person-Centred Care: A Theory-Driven Approach Based on the Normalization Process Theory). Field notes were taken during and immediately after each session to document group dynamics, contextual factors, and salient non-verbal cues relevant to data interpretation. Each FGD lasted approximately 45–60 min.

**Table 1:** Semi-structured interview guide on AI- and robotics-supported person-centred care: a theory-driven approach based on the normalization process theory

Section	Purpose / Theoretical Axis (NPT Construct)	Sample Questions
A. Meaning-Making (Coherence)	Explore how participants conceptualize and make sense of AI- and robotics-supported person-centred care in nursing and midwifery education.	• What does “AI- and robotics-supported person-centred care” mean to you? • How do you make sense of this concept in relation to your educational experiences and professional identity? • In your view, how does this technology influence the essence of nursing or midwifery? Does it bring benefits, risks, or both? • How do you see its relationship with the core values of nursing education (respect, autonomy, privacy, justice)? • Can you provide an example of how these values can be protected in a technologically enhanced environment?
B. Engagement and Ownership (Cognitive Participation)	Examine how participants and institutions adopt, legitimize, and sustain the integration of AI and robotics into education.	• Who or which groups within your faculty are leading or resisting the digital transformation process? • What do you think underlies these attitudes? • What role do you see yourself playing in this process (initiator, supporter, observer, skeptic)? • Which stakeholders (e.g., clinical educators, IT units, ethics committees, hospital administration) should be involved for sustainable implementation? • How could collaboration and trust among these stakeholders be established? • How important is inter-institutional communication and trust in this process?
C. Implementation and Operation (Collective Action)	Explore how AI and robotics are practically embedded in curricula, teaching, and institutional processes.	• In which courses or semesters should AI/robotic content be placed, and on what prior knowledge or skills should it build? • Have you had any relevant teaching experiences supporting this integration? • Which instructional methods (e.g., case studies, simulations, projects) do you find most effective? • How would you design a curriculum that enables students to achieve similar learning outcomes in classroom, simulation, and clinical settings? • Can you describe a small example of such a curriculum plan? • How should coordination among teaching staff be ensured? • What ethical or emotional safeguards should be integrated into the design to prevent the loss of human connection? • What communication, empathy, or reflection-based practices could support this balance? • What was the most concrete challenge you faced during this process (e.g., infrastructure, data access, ethics, workload)? • How might you solve such an issue under limited resources? • What was the most challenging yet instructive experience for you in this transformation?
D. Monitoring and Evaluation (Reflexive Monitoring)	Understand how participants assess educational impact, student outcomes, and ethical dimensions of AI/robotics integration.	• How do you observe or measure students’ ability to critically evaluate AI-based outcomes and make person-centred clinical decisions? • Can you provide an example of an evaluation criterion reflecting this observation? • Do you think such measures also capture students’ ethical awareness? • How would you define “success” in AI/robotic education? • Which types of evidence (e.g., OSCE, portfolio, case reflections, mini-CEX) best demonstrate success? • Should these measures reflect only technical proficiency or also holistic care values? • Over the past year, what have you chosen to start, stop, or continue in your AI/robotics teaching practices? • What experiences influenced these decisions? • How have you observed the effects of these decisions on students or colleagues?
E. Turning Points and Roadmap (Future Outlook)	Identify transformative experiences and future directions for sustainable integration.	• Can you share a specific event or experience related to AI or robotics that significantly challenged or shifted your perspective? (e.g., student reaction, ethical dilemma, technical failure, unexpected learning outcome) • What decisions did you make at that moment, and how might you approach it differently now? • Why was that moment meaningful to you, and what did it change in your thinking? • Looking ahead to the next year, what are two or three concrete actions you would like to take to strengthen AI/robotics education in your courses or faculty? • Why are these steps important to you? • If you had unlimited resources and support, where would you start this transformation? • What structural change would you prioritize first (e.g., upgrading simulation labs, launching partnerships, adding ethics modules)?
Closure	Ensure ethical debriefing and participant agency.	• Is there anything you would like to add that we haven’t discussed? • Would you like to receive a summary of your interview findings?

### Data analysis

The qualitative data were analyzed using conventional content analysis, following established methodological guidance in qualitative health research [24]. To ensure confidentiality, all participants were assigned anonymized identifiers (e.g., P1, A26, F). All audio-recorded FGDs were transcribed verbatim by two members of the research team who were not involved in moderating the sessions; both transcribers had prior training and experience in qualitative transcription and analysis, ensuring accuracy and consistency. The analysis followed an inductive–deductive strategy. First, an inductive approach was used to allow codes and preliminary categories to emerge directly from the data. Two researchers independently conducted line-by-line open coding to identify meaning units [25], and MAXQDA 24 software was used to organize codes, compare segments, and cluster related meaning units. Coding was iterative and involved constant comparison across transcripts, with discrepancies resolved through discussion and consensus. Inductively derived categories were subsequently refined into broader themes, which were then interpreted through the NPT constructs and synthesized into higher-order pedagogical dimensions forming the basis of the ACPM. In the second phase, a deductive analytical lens based on the four core constructs of NPT coherence, cognitive participation, collective action, and reflexive monitoring [19] was applied to systematically interpret how participants made sense of, engaged with, operationalized, and evaluated AI- and robotics-supported person-centred care within educational contexts. The integration of inductive and deductive approaches enhanced interpretive rigor and theoretical depth, ensuring that the findings were both grounded in participants’ experiences and aligned with the NPT framework, thereby offering a comprehensive understanding of how complex technological innovations become embedded in nursing and midwifery education. As analytic patterns across inductively derived categories were examined through the NPT constructs, these analytical linkages were progressively synthesized into higher-order pedagogical dimensions. This analytic synthesis informed the development of the ACPM, with the alignment between NPT constructs and emergent pedagogical dimensions presented in Table 2 (Table 2. Alignment Between NPT Constructs and the ACPM).

**Table 2:** Alignment between normalization process theory (NPT) constructs and the augmented caring pedagogy model (ACPM)

NPT Construct	Description	ACPM Component	Pedagogical Function in Education
Coherence	Sense-making of a technological innovation development of a shared cognitive understanding	Human Technology Synergy (Augmented Caring Lens)	Integration of AI-generated outputs with person-centred values, empathy, therapeutic communication, and relational care
Cognitive Participation	Engagement and commitment of key actors collective ownership of the innovation	Collaborative Integration Loop	Interdisciplinary collaboration – shared responsibility among faculty, pharmacists, and informatics specialists in AI-supported education
Collective Action	Enactment of the innovation in everyday practice, mobilization of institutional resources	Institutional Anchoring Cycle	Standardization and institutionalization of AI-supported processes, barcode systems, robotic dispensing, and the “seven rights” of medication administration
Reflexive Monitoring	Ongoing appraisal and evaluation reconfiguration of practices over time	Reflective Adaptation Cycle	Continuous performance monitoring, ethical feedback, reflective learning, and iterative improvement are supported by AI-based analytics

#### Rigour and reflexivity

To ensure the study’s trustworthiness, strategies addressing credibility, dependability, confirmability, and transferability were applied throughout the research process [21]. Credibility was strengthened through prolonged engagement with the data, iterative analysis guided by both inductive and NPT-informed deductive processes, and peer debriefing among the research team to refine interpretations. Additionally, member checking was conducted with selected participants to verify the accuracy and resonance of preliminary themes. Dependability was ensured by maintaining a comprehensive audit trail in MAXQDA 24, including coded transcripts, analytic memos, and decision logs, thereby enhancing transparency and replicability. Confirmability was achieved through reflexive journaling, in which researchers systematically documented their assumptions, positionalities, and potential biases. These reflections were reviewed during regular research team meetings to minimize subjective influence on data interpretation. Transferability was addressed by providing rich, contextualized descriptions of participants’ demographic characteristics, institutional contexts, and experiences related to the integration of AI and robotics into person-centred fundamental care education, allowing readers to assess the applicability of findings to other contexts. Reflexivity was a central component of the research process.

The researchers’ dual roles as nursing and midwifery educators and as qualitative researchers were explicitly acknowledged and critically examined. These professional roles facilitated rapport and trust during focus group discussions, as participants perceived the researchers as peers with shared disciplinary knowledge. However, the researchers also recognized that this positionality could influence data collection by fostering assumptions of shared understanding or encouraging socially desirable responses. To mitigate these risks, the researchers employed open-ended questioning, avoided evaluative or affirming responses, and actively encouraged participants to articulate diverse and critical perspectives. During data analysis, the researchers remained attentive to the potential for interpretive bias arising from their prior pedagogical experiences and commitments to person-centred care, as well as the integration of AI and robotics in health education. Reflexive journaling was used to make preconceptions explicit, and emerging interpretations were regularly discussed within the research team. Disconfirming cases were actively sought, and analytic decisions were revisited to ensure that themes were grounded in participants’ accounts rather than researchers’ professional assumptions. This ongoing reflexive engagement enhanced analytic rigour and supported a theoretically informed yet empirically grounded interpretation of findings within the NPT framework. The researchers critically reflected on their dual roles as nursing and midwifery academics and as investigators, acknowledging how their professional experiences and values could shape the interpretation of findings. This continuous reflexive stance further enhanced the study’s interpretive depth and strengthened its theoretical alignment with the NPT framework.

## Results

### Characteristics of participants

Twenty faculty members from nursing and midwifery departments participated, drawn from public (*n* = 11, 55%) and foundation (*n* = 9, 45%) universities across nine institutions. Most participants were female (*n* = 16, 80%), and the group was evenly split between nursing and midwifery (each *n* = 10, 50%). Academic ranks ranged from research assistant to professor. Mean teaching experience was 10 ± 7.69 years (1–25), and mean clinical experience was 15 ± 7.93 years (3–26). All taught clinical/practical courses in the past two years, and most also taught theoretical (*n* = 18, 90%) and simulation-based sessions (*n* = 14, 70%). Over half had attended AI/robotics-related training (*n* = 11, 55%), while reported use of AI/robotics in teaching was mostly rare or occasional. Perceived institutional AI policies were mixed (50% yes), rubric use for person-centred care assessment varied, and technical infrastructure/support was rated moderate (mean = 2.85 ± 1.13; Table 3. Descriptive characteristics of participants (n:20)).

**Table 3:** Descriptive characteristics of participants (n:20)

Variables	*n*	%
Gender		
Female	16	80
Male	4	20
University Type		
Public	11	55
Foundation	9	45
Field of Expertise		
Nursing	10	50
Midwifery	10	50
Academic Title		
Research Assistant	4	20
Lecturer	3	15
Assistant Professor	5	25
Associate Professor	5	25
Professor	3	15
Types of Courses Taught (past 2 years) ^*^		
Theoretical	18	90
Clinical/Practical	20	100
Simulation	14	70
Receipt of AI/Robotics-Related Trainings, Workshops, or Certificates (past 2 years)		
Yes	11	55
No	9	45
Use of AI/Robotics in Your Courses		
Never	1	5
Rarely	10	50
Sometimes	7	35
Frequently	2	10
Perceived Existence of Institutional Guidelines/Ethical Policies on AI		
Yes	10	50
No	3	15
Don’t know	7	35
Do you have a written rubric for assessing person-centred care teaching?		
Yes	7	35
No	7	35
Don’t know	6	30
Variables	Mean ± SD	Min.-Max.
Age (years)	39 ± 8.40	27–52
Teaching Experience (years)	10 ± 7.69	1–25
Clinical Experience (total years)	15 ± 7.93	3–26
Perceived Adequacy of Technical Infrastructure/Support	2.85 ± 1.13	1–5

^*^Multiple answers were allowed for this question

*SD* Standard deviation

### Themes and sub-themes

The qualitative analysis identified four interrelated themes and 12 subthemes that describe how nursing and midwifery faculty perceived, engaged with, and evaluated the integration of AI and robotics into person-centred education (Table 4). Guided by NPT, the themes were organized to reflect participants’ accounts related to coherence (sense-making), cognitive participation (engagement), collective action (implementation), and reflexive monitoring (appraisal of experiences and perceived effects).

**Table 4:** Thematic framework of faculty perspectives on AI- and robotics-supported person-centred care in nursing and midwifery education

Main Theme	Subtheme	Related NPT Construct	Description / Focus Area	Example Quotations
Theme 1. Making Sense of AI- and Robotics-Supported Person-Centred Care	*1.1 Technology as a Tool for Professional Advancement*	Coherence *Communal Specification*	Viewing technology as a catalyst for modernization, professional prestige, innovation, and the advancement of patient-centred, safe, and high-quality care in nursing and midwifery education.	● “Midwifery is no longer behind the times; it’s becoming a profession that uses and explores technology.” (P4, A44, F). ● “These technologies help us provide safer, more accurate, and more person-centred care. Especially in surgical procedures, robotic systems reduce infection risks and ensure more consistent outcomes.” (P11, A39, M).
	*1.2 Emotional and Ethical Tensions*	Coherence *Individual Specification*	Concerns about the erosion of empathy, compassion, and human connection through technological reliance.	● “AI and robotics are very useful, but they can’t teach empathy. Students need to learn to look at the person, not just the screen, patients need empathy more than robots.” (P2, A47, F) ● “When we provide care through robots, the warmth disappears; sometimes even a simple touch is a form of healing.” (P3, A38, F).
	*1.3 Reconciling Digital Systems with Person-Centred Values*	Coherence *Communal Appraisal*	Balancing digital efficiency with ethical values such as privacy, justice, and patient autonomy.	● “In AI-based patient record systems, it’s not always clear who owns the data. That makes us anxious.” (P5, A38, F). ● “In some institutions, there are almost no mechanisms for ethical guidance in AI or robotic care. Everyone acts according to their own conscience.” (P11, A39, M).
	*1.4 Transformation of Teaching Philosophy*	Coherence *Internalization*	Redefining teaching as facilitation of critical thinking and ethical awareness through technology.	● “We used to just lecture; now, with AI-based scenarios, students create their own solutions.” (P4, A44, F) . ● “Before, we only showed scenarios, but now students take an active role within the simulation.” (P19, A27, F).
Theme 2. Engaging with Technological Transformation	*2.1 Generational and Cultural Differences*	Cognitive Participation *Initiation*	Differences in digital readiness and openness among faculty shaped by age and teaching culture.	● “Younger instructors use technology easily and adapt quickly. Our generation is a bit more distant.” (P20, A29, F). ● “Some colleagues still prefer traditional methods; for them, digital transformation is quite challenging.” (P12, A45, M).
	*2.2 Motivation*,* Leadership*,* and Systemic Resources*	Cognitive Participation *Enrolment & Activation*	Leadership, infrastructure, and resources as key enablers of engagement and motivation.	● “When the administration is supportive, everything moves faster, but most of the time, the infrastructure is lacking.” (P4, A44, F). ● “If new projects don’t get funding, everything remains at the discussion level; we can’t move to implementation.” (P13, A39, F).
	*2.3 Shared Ownership and Collaborative Culture in Education*	Cognitive Participation *Legitimation*	Promoting teamwork, shared vision, and institutional responsibility for sustainable transformation.	● “Students are ahead of us in these areas; their enthusiasm affects us too.” (P3, A38, F). ● “You can’t do it alone; progress only lasts when it’s a team effort.” (P10, A29, F).
Theme 3. Operationalizing and Sustaining AI Integration	*3.1 Interdisciplinary Collaboration*	Collective Action *Interactional Workability*	Cross-disciplinary cooperation between health, informatics, and engineering faculties.	● “AI is not something we can handle alone; progress is impossible without engineering support.” (P3, A38, F). ● “We know the clinical side, but for the software part, we definitely need to work with the informatics team.” (P13, A39, F).
	*3.2 Standardization and Quality Assurance*	Collective Action *Contextual Integration*	Need for standardized ethical frameworks and quality mechanisms across faculties.	● “Every faculty applies it differently; we need to establish a standard.” (P10, A29, F). ● “The ethical framework should be clear; otherwise, practices become confusing.” (P5, A38, F).
Theme 4. Learning Outcomes, Equity, and Ethical Responsibility in AI-Enhanced Education	*4.1 Observed Learning Outcomes and Students’ Self-Assessment*	Reflexive Monitoring *Systematization*	Enhanced self-awareness, reflection, and emotional intelligence through AI-based learning.	● “In the AI system, students notice their mistakes immediately, which makes them more careful.” (P6, A38, F) ● “AI-based simulations help students review what they’ve done. It’s a great preparation for real patient care.” (P10, A29, F).
	*4.2 Achievement*,* Equity*,* and Evaluation Approaches*	Reflexive Monitoring *Communal Appraisal*	Data-driven assessments improving fairness, with concerns about empathy being overlooked.	● “AI-supported assessment allows us to measure student performance more objectively.” (P12, A45, M). ● “Before, grading could sometimes be intuitive; now the system provides measurable, clear, and objective data.” (P5, A38, F).
	*4.3 Ethical Responsibility and Sustainability Perspective*	Reflexive Monitoring *Reconfiguration*	Data security, equal access, and ethical boundaries as the foundation for sustainable AI use.	● “Data security is a very sensitive issue students must also be made aware of.” (P8, A45, F). ● “Some universities have plenty of resources while others have none. This inequality could grow in the future.” (P13, A39, F).

#### Theme 1. Making sense of AI- and robotics-supported person-centred care

Participants described how they made sense of AI and robotic technologies in relation to person-centred care in nursing and midwifery education. Their accounts addressed the perceived purpose and value of these technologies, the tensions they raised, and related teaching practices.

##### Subtheme 1.1: Technology as a tool for professional advancement

Participants described AI and robotics as supporting modernization and professional visibility in nursing and midwifery, including innovation, academic productivity, and clinical safety:

*“Midwifery is no longer behind the times; it’s becoming a profession that uses and explores technology.” (P4*,* A44*,* F)*.

Participants highlighted interdisciplinary collaboration and the enhanced precision afforded by AI-supported systems, particularly in high-risk or procedural contexts:

*“These technologies help us provide safer*,* more accurate*,* and more person-centred care.” (P11*,* A39*,* M)*.

##### Subtheme 1.2: Emotional and ethical tensions

Participants also raised concerns about potential reductions in empathy, emotional connection, and the “human touch” in care, noting that AI may not replicate relational aspects central to person-centred care:

*“Students need to learn to look at the person*,* not just the screen; patients need empathy more than robots.” (P2*,* A47*,* F)*.

Several participants noted that emotional neutrality could both support and limit care:

*“AI reduces errors*,* but it might also reduce the emotional connection essential to care.” (P9*,* A32*,* F)*.

##### Subtheme 1.3: Reconciling digital systems with person-centred values

Participants raised questions about privacy, data ownership, autonomy, justice, and accountability in AI-enhanced care environments:

*“In AI-based patient record systems*,* it’s not always clear who owns the data.” (P5*,* A38*,* F)*.

They emphasized the need for institutional policies and ethical guidelines to support safe and equitable use:

*“There should be an accessible institutional policy on how these technologies are used.” (P20*,* A29*,* F)*.

##### Subtheme 1.4: Transformation of teaching philosophy

Participants described changes in teaching practices associated with AI-supported tools, including problem-solving, active learning, and student autonomy:

*“It used to be one-way; now students can ask questions to the AI*,* which deepens their learning.” (P11*,* A39*,* M)*.

They also described a shift toward facilitation in teaching:

*“We’re no longer just explaining; we’re guiding the learning process.”* (P9, A32, F).

#### Theme 2. Engaging with technological transformation

Participants described factors that influenced their engagement with AI- and robotics-supported practices, including attitudes toward change, institutional support, and available resources.

##### Subtheme 2.1: Generational and cultural differences

Participants described differences in attitudes toward technological transformation by age and digital experience, reporting that younger academics were generally more open to innovation, whereas senior faculty were more cautious or resistant:

*“Some colleagues still prefer traditional methods; for them*,* digital transformation is quite challenging.”* (P12, A45, M).

Several faculty members pointed out that these differences were not only age-related but also shaped by long-standing educational habits and institutional culture:

*“Resistance to technology is more about habit. It’s not easy for someone who has been teaching the same way for years to change.”* (P5, A38, F).

##### Subtheme 2.2: Motivation, leadership, and systemic resources

Participants described institutional support and leadership as influencing engagement, and reported that limited infrastructure, technical support, and bureaucratic barriers constrained implementation:

*“When the administration is supportive*,* everything moves faster*,* but most of the time*,* the infrastructure is lacking.”* (P4, A44, F).

Many participants highlighted the role of institutional leadership in facilitating engagement:

*“When the faculty administration takes ownership*,* everyone gets motivated; if there’s no top-level direction*,* no one continues on their own.”* (P6, A51, F).

They also reported that inadequate technical infrastructure and limited trained personnel constrained sustainable implementation:

*“When there’s no technical support*,* we can’t even open the system sometimes*,* and it puts us in a difficult position in front of students.”* (P12, A45, M).

##### Subtheme 2.3: Shared ownership and collaborative culture in education

Faculty members stressed that sustainable technological transformation relies on collective engagement, with students’ enthusiasm and a shared team vision strengthening motivation and continuity:

*“When we create a shared vision within the faculty*,* everyone’s contribution increases.”* (P14, A45, F).

Several faculty members noted that collaborative projects and working groups played a significant role in developing this sense of ownership:

*“We organized AI-themed seminars where everyone shared ideas. It helped us build a common understanding.”* (P8, A45, F).

#### Theme 3. Operationalizing and sustaining AI integration

Participants described how AI and robotics were operationalized in education, including collaboration practices and reported challenges related to implementation and sustainability.

##### Subtheme 3.1: Interdisciplinary collaboration

Participants described the need for cross-disciplinary collaboration (e.g., engineering, informatics, and health sciences) when developing AI- and robotics-supported projects and educational platforms:

*“We know the clinical side*,* but for the software part*,* we definitely need to work with the informatics team.”* (P13, A39, F).

They also described perceived benefits of working across disciplines for strengthening both content and technology:

*“When we work across disciplines*,* both the content and the technology become much stronger.”* (P1, A32, F).

##### Subtheme 3.2: Standardization and quality assurance

Participants reported that technological practices varied across faculties, which they associated with differences in instructional quality. They referred to the need for standardized protocols, ethical guidelines, and quality monitoring mechanisms to support consistency and equity in educational delivery:

*“The ethical framework should be clear; otherwise*,* practices become confusing.”* (P5, A38, F).

*“If there’s no quality assurance*,* educational inequality emerges.”* (P9, A32, F).

Some participants also referred to the value of a coordinated approach at the national level:

*“Instead of each university applying its own methods*,* there should be a national strategy.”* (P7, A30, F).

#### Theme 4. Learning outcomes, equity, and ethical responsibility in AI-enhanced education

Participants described perceived learning outcomes, equity-related considerations, and ethical responsibilities associated with AI- and robotics-enhanced education (Table 5. Full Operationalization of the ACPM Aligned with NPT: A High-Fidelity AI- and Robotics-Supported Medication Safety Education Scenario).

**Table 5:** Full operationalization of the augmented caring pedagogy model (ACPM) aligned with normalization process theory (NPT): A High-Fidelity AI- and Robotics-Supported medication safety education scenario

Phase (NPT)	ACPM Component	Pedagogical Objective	Detailed Educational Implementation	Reflective, Ethical, and Professional Emphases
Phase 1 – Coherence (Sense-Making)	Human–Technology Synergy (Augmented Caring Lens)	To enable students to make sense of both the functional role of technology and its relationship to human-centred care	An AI system generates an alert indicating a potential drug–drug interaction during the preparation of medication. The educator explicitly asks the following reflective questions: *“How did this alert support your clinical decision-making?”* and *“At which point did your professional judgement become essential?”* Through facilitated discussion, students reinterpret technology not as a decision-maker, but as a tool that strengthens clinical reasoning and patient safety.	Empathic communication, therapeutic touch, and patient trust are emphasized as the non-negotiable core of person-centred care, reinforcing that AI can augment but never replace human presence.
Phase 2 – Cognitive Participation (Engagement and Ownership)	Collaborative Integration Loop	To cultivate collective responsibility, interdisciplinary collaboration, and institutional ownership in technology integration	The simulation scenario is co-designed by nursing or midwifery faculty members, pharmacists, and informatics specialists. Students are assigned rotating roles within the simulation, including bedside care provider, documentation and verification officer, and AI system operator. Through this role distribution, students experience technology as something they collectively sustain rather than passively use.	In alignment with NPT, this phase represents the innovation legitimation process, whereby technology shifts from an individual tool to a shared institutional responsibility.
Phase 3 – Collective Action (Operationalization and Implementation)	Institutional Anchoring Cycle	To operationalize AI and robotic technologies within routine care processes and embed them into standardized institutional workflows	A robotic medication dispensing system is integrated into the scenario, automatically verifying correct medication and dosage. Students compare AI-assisted verification with the traditional “seven rights” of medication administration. Barcode verification is a mandatory step, and the AI system records any omitted or incomplete safety checks. During the scenario, the AI system generates a contextual alert: *“Time verification was not completed. What could be the potential clinical consequences of this omission?”*	This phase exemplifies the contextual integration dimension of NPT, illustrating how technology becomes embedded within institutional routines rather than functioning as an external or optional add-on.
Phase 4 – Reflexive Monitoring (Evaluation and Continuous Adaptation)	Reflective Adaptation Cycle	To support the continuous evaluation of student performance, ethical awareness, and adaptive professional learning	At the conclusion of the scenario, the AI system generates a structured, reflective performance report detailing omitted safety steps, the accuracy of clinical decision-making, ethical risk awareness, and indicators of empathetic communication. Students then engage in guided high-level reflective questioning, including: *“At which point did AI strengthen your clinical intuition?” “Where did you recognize the limitations of AI and rely on professional judgement?”* and *“Which aspects of empathic care were supported or challenged by technology?”*	This phase corresponds to the reconfiguration stage of NPT and underscores that AI–robotic integration represents not merely a technical innovation but an epistemological, ethical, and pedagogical transformation. Ethical awareness, person-centred values, and technological competence are strengthened simultaneously.

##### Subtheme 4.1: Observed learning outcomes and students’ self-assessment

Participants reported that AI- and robotics-supported technologies were associated with students’ self-monitoring, critical thinking, and ethical sensitivity in scenario-based learning:

*“In the AI system*,* students notice their mistakes immediately*,* which makes them more careful.”* (P6, A38, F).

Some participants also referred to communication-related learning in robotic applications:

*“Through robotic applications*,* students also realize their communication mistakes; it helps strengthen the human side of care.”* (P9, A32, F).

Participants further described AI-based simulations as enabling exposure to clinical situations and patient profiles that students may not encounter during routine placements, including examples involving vulnerable or marginalized groups:

*“Students can experience how to initiate and maintain communication or provide care for LGBTQ+ patients*,* even if they don’t meet such cases in the clinic. AI-based simulations make these learning moments possible.”* (P12, A45, M).

This example was mentioned by a small number of participants and is presented here as an illustrative account of perceived learning opportunities in simulation-based education.

##### Subtheme 4.2: Achievement, equity, and evaluation approaches

Participants described AI- and robotics-supported tools as influencing assessment practices, including increased use of data-driven and standardized evaluation approaches:

*“AI-supported assessment allows us to measure student performance more objectively.”* (P12, A45, M).

Some participants noted concerns that such systems may not capture all individual characteristics:

*“Everything has become measurable*,* but sometimes aspects like emotional intelligence or empathy are left out.”* (P18, A32, F).

Participants also described these tools as potentially reducing subjectivity in evaluation, including in multicultural and gender-diverse classrooms:

*“The system doesn’t judge who you are or how you speak*,* it only evaluates the performance. This gives students who are quieter or less confident an equal chance.”* (P17, A42, F).

At the same time, some participants highlighted that equalized cases do not eliminate contextual differences among students:

*“Even if AI gives everyone the same case*,* not every student starts from the same place*,* access to digital literacy and language skills still matters.”* (P20, A29, F).

##### Subtheme 4.3: Ethical responsibility and sustainability perspective

Participants emphasized ethical responsibility, data security, and equitable access as considerations for long-term use of AI and robotics in education:

*“Data security is a very sensitive issue*,* students must also be made aware of.”* (P8, A45, F). *“Some universities have plenty of resources while others have none. This inequality could grow in the future.”* (P13, A39, F).

They also referred to the role of ethical guidance and oversight for future implementation:

*“In the future*,* these technologies must be under ethical supervision; otherwise*,* it could turn into an uncontrolled field.”* (P17, A42, F).

## Discussion

The findings illustrate how nursing and midwifery faculty engage with the pedagogical, ethical, and institutional demands of integrating AI and robotics into person-centred education. Rather than treating these technologies as discrete tools, participants described them as shaping teaching practices, professional roles, and responsibilities, while also raising concerns about empathy, accountability, equity, and uneven institutional readiness. Interpreted through NPT, these accounts indicate an ongoing process of sense-making, collective engagement, enactment, and appraisal in educational settings, which informed the development of the ACPM as a framework linking person-centred values with institutional conditions and technology-enabled practices.

These findings should be interpreted within the Turkish higher education and healthcare context, characterized by centrally regulated academic governance under the Council of Higher Education (CoHE) and recent national efforts to transform higher education [26]. In Türkiye, curricula are largely shaped within national frameworks, while institution-level initiatives operate within overarching regulations; at the time of data collection, explicit pedagogical guidance and standardized regulatory frameworks for AI/robotics integration in health professions education were still evolving. This may help explain variation in institutional readiness, reliance on individual faculty initiative, and the prominence of ethical uncertainty and sustainability concerns. In the absence of clear national standards, educators may therefore be navigating both pedagogical innovation and ethical responsibility.

### Redefining AI- and robotics-supported person-centred care: Augmented Caring Pedagogy

**Fig. 1:**
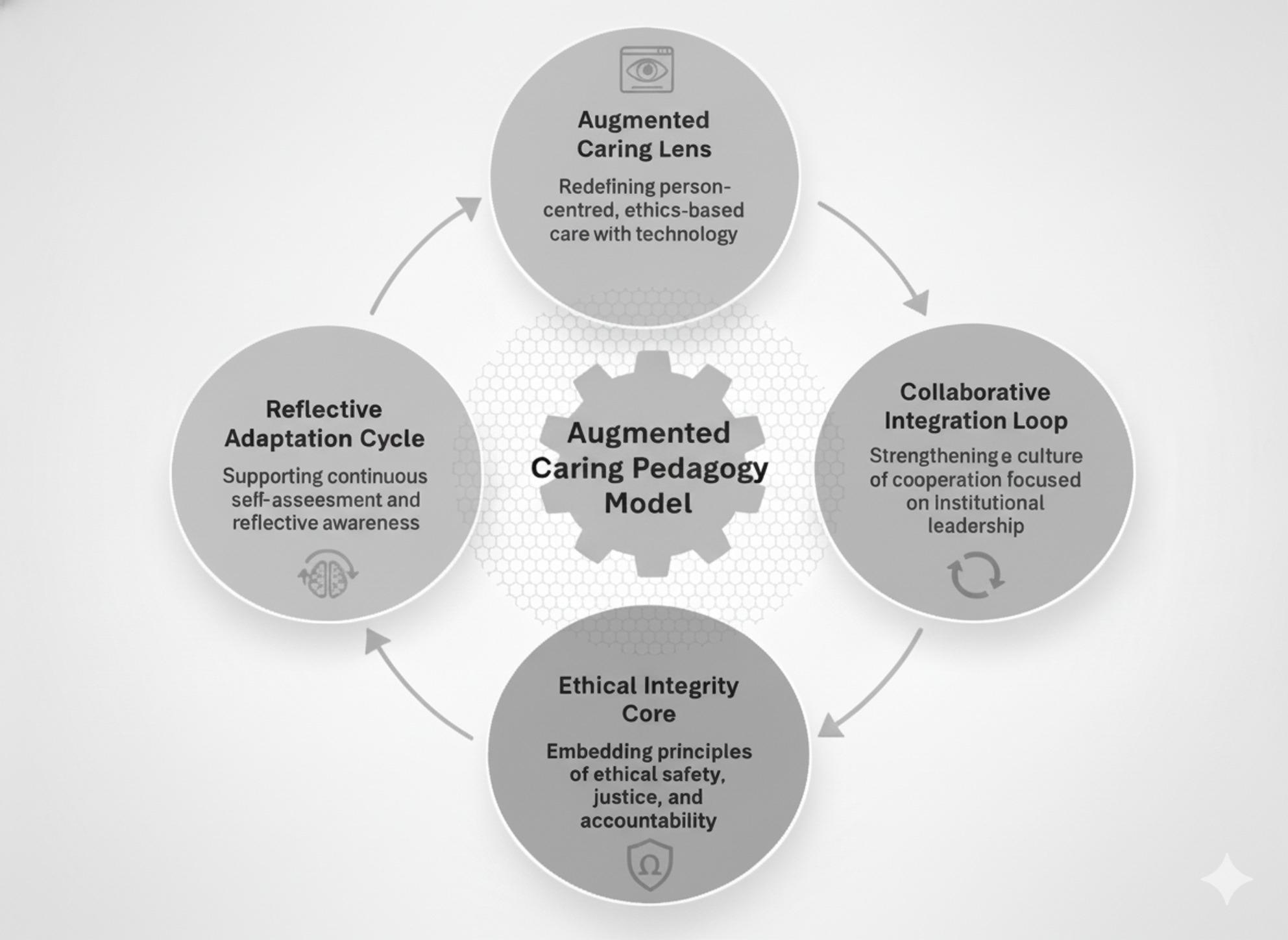
Structure of augmented caring pedagogy model

As AI and robotic technologies increasingly enter nursing and midwifery education, there is a growing need to reconceptualize person-centred care in ways that integrate technological innovation with humanistic and ethical pedagogical principles [27]. In this study, participants’ accounts suggest a pedagogical shift that extends beyond incremental technical adoption: AI and robotics were discussed as shaping how person-centred care is conceptualized, taught, and enacted within educational practice. This perspective adds to a literature that has largely emphasized efficiency and accuracy [1, 4, 5, 28] by highlighting perceived implications for teaching philosophy, professional roles, and moral responsibility. At the same time, participants’ descriptions of both benefits and ethical tensions indicate that while AI may support clinical reasoning, safety, and learner autonomy, educators may also need to deliberately attend to empathy, relational presence, and accountability through intentional pedagogical design. Consistent with evidence that simulation-based learning can strengthen empathy [29], our findings suggest that empathy is not inherently incompatible with AI-mediated learning but may require explicit cultivation within technology-enhanced environments. Recent work likewise underscores that emotional intelligence and critical thinking remain central to person-centred nursing education in technology-enhanced contexts, aligning with participants’ concerns that AI should complement rather than replace humanistic care values [30]. This discussion is particularly salient in Türkiye, where a strong tradition of caring coexists with digitalized health record infrastructures such as e-Nabız [31]. This convergence suggests the importance of safeguarding culturally rooted “human touch” values (e.g., compassion and tenderness) within educational practice, even where technical readiness for digital transformation is high [32]. Interpreted through NPT, this reflects coherence as a process of aligning digital innovation with professional values, informing the ACPM component of Human–Technology Synergy (Fig. 1).

### Technological engagement and institutional ownership in education: collaborative integration loop

Faculty members’ engagement with AI and robotic technologies in education can be understood as shaped by institutional conditions, including governance, leadership, and shared pedagogical ownership, rather than individual motivation alone. This interpretation is consistent with evidence that digital transformation in health education requires cultural readiness, institutional governance, and sustained strategic planning beyond tool adoption [1, 7, 33]. Related research also suggests that work environment quality and professional well-being can influence educators’ capacity to sustain AI-supported practices over time [34].

Within NPT, these dynamics map onto cognitive participation and legitimation, as normalization is supported when innovations are institutionally endorsed and collectively enacted within organizational networks [19, 35]. In this sense, academics may function not only as end users but also as stakeholders shaping the pedagogical architecture of AI-enhanced education [8, 11]. Accordingly, barriers such as limitations in infrastructure, funding, ethical guidance, and professional development can be interpreted as constraints on institutional readiness rather than individual deficits [12, 36, 37]. This need for institutional ownership is salient in Türkiye in the context of expanding nursing student quotas, and similar capacity pressures internationally can strain faculty-to-student ratios and the quality of clinical supervision [38]. Under such conditions, describing AI as a “supportive tool” may reflect pragmatic efforts to manage large cohorts while maintaining person-centred standards, consistent with discussions of AI as a “teaching assistant” to reduce workload and mitigate burnout. As a contribution, the ACPM Collaborative Integration Loop (Fig. 1) frames sustainable AI integration as dependent on collective participation, institutional support, and shared ownership, offering a pathway from episodic initiatives to normalized educational practice.

### Operationalizing and sustaining AI and robotic integration: interdisciplinary collaboration and standardization

Theme 3 highlights that sustaining AI and robotics in health professions education may depend on institutional capability and coordination, rather than isolated teaching initiatives. Evidence that standardized and well-supported technology-enhanced training is associated with improved learner outcomes (e.g., satisfaction and self-confidence) supports the importance of coordination and continuity in implementation [39]. Prior work likewise emphasizes the value of building educators’ technical competencies and institutional capacity for scaling AI-enhanced pedagogy [8, 11], and the role of structured collaboration across engineering and health sciences in enabling durable integration [28]. In addition, calls for ethical frameworks, equitable access policies, and clear implementation guidance underscore the role of governance and standardization in maintaining educational quality [12, 16]. Together, these points align with arguments that institutionalization is supported by organizational ownership and multisectoral stakeholder alignment, beyond individual enthusiasm alone [1, 8]. From an NPT perspective, this theme elaborates collective action by specifying practical requirements for enactment in educational settings, including resource mobilization, role clarification, and integration into governance and quality assurance structures. As practical implications, we propose three system-level levers to support normalization beyond pilot projects: Joint AI–Education Laboratories, National and Institutional Standards, and Continuous Professional Development. These levers provide an empirical rationale for the ACPM Institutional Anchoring Cycle (third component), which frames sustainability as embedded in routine workflows, resourcing, and ethically informed oversight rather than episodic, technology-driven initiatives.

### Learning outcomes, equity, and ethical responsibility: Reflective Adaptation Cycle

Theme 4 suggests that AI and robotics may support continuous learning, self-assessment, and ethical reflection in nursing and midwifery education, beyond their function as instructional tools. Participants’ accounts, together with prior evidence, indicate that technology can support feedback processes for performance monitoring and structured reflection, aligning with findings that AI-enabled simulations may enhance self-awareness and learning satisfaction [8, 11, 39] and that robotic applications may foster reflective thinking and strategic decision-making [28]. These observations map onto NPT’s reflexive monitoring, in which innovations are sustained through ongoing observation, evaluation, and recalibration of practice over time [19, 35]. Accordingly, AI may be better framed as part of an evolving learning environment that informs iterative pedagogical improvement rather than a static add-on. Evidence from comparable high-stress educational contexts also suggests that reflective capacity and ethical sensitivity are shaped by psychosocial and structural conditions, rather than by technology alone. For example, nursing students in Palestinian clinical training settings reported high perceived stress alongside moderate resilience, suggesting that contextual stressors can influence engagement, reflection, and ethical responsiveness [30]. Similarly, evidence-based practice competency research indicates that cognitive readiness and ethical reasoning are closely tied to educational support structures, particularly in resource-constrained environments [40]. Equity becomes salient as assessment and feedback are mediated by technology: access to tools does not necessarily translate into equitable learning benefits. Therefore, technology-enabled assessment may introduce ethical and governance needs, including guidance on ethical use, algorithmic transparency, data security, and policies to support equitable access. These considerations inform the ACPM Reflective Adaptation Cycle (fourth component; Fig. 1), which frames technology integration as an ongoing process of quality improvement grounded in self-assessment and ethically informed oversight. Building on this operationalization, broader ACPM implications for nursing and midwifery education and practice (e.g., curriculum design, faculty development, governance, teaching practice, clinical application, and equity) are summarized in Table 6.

**Table 6:** Implications of the augmented caring pedagogy model (ACPM) for nursing and midwifery education and practice

Domain	ACPM Component	Key Focus	Practical Implications
Curriculum Design	Human–Technology Synergy (Augmented Caring Lens)	Embedding ethical and human-centred AI competencies	Educators can integrate AI-supported learning activities without compromising humanistic values. Simulation scenarios may include ethical reasoning tasks, empathy-enhancing interactions, and human–technology communication skills. ACPM can serve as a curricular roadmap ensuring that digital competencies and caring principles progress in parallel rather than in isolation.
Faculty Development	Collaborative Integration Loop	Building institutional readiness and shared ownership	Nursing and midwifery schools can establish technology champions or faculty AI leads, create interdisciplinary working groups with engineering and informatics, and allocate protected time for faculty development in AI-supported pedagogies. These strategies reduce resistance, build confidence, and foster a shared culture of innovation.
Standardization and Governance	Institutional Anchoring Cycle	Strengthening ethical, quality, and governance frameworks	Institutions can use ACPM to develop minimum standards for AI-supported simulation and assessment, establish clear guidelines for data security, algorithmic transparency, and academic integrity, and implement quality assurance processes that monitor equity and learning outcomes across student groups.
Teaching Practice	Reflective Adaptation Cycle	Fostering reflective and adaptive learning	Educators can incorporate AI-driven feedback tools to support students’ self-evaluation, reflective journaling, and the development of clinical reasoning. Real-time performance analytics may be used to tailor instruction and identify learning needs early, promoting adaptive and individualized learning environments.
Clinical Practice	Cross-cutting ACPM principles	Enhancing decision-making and patient safety	ACPM guides clinical educators and preceptors in using AI as a complement—not a substitute—for professional judgement. Educational strategies include teaching students to critically appraise algorithmic outputs, apply AI-based early warning systems, and reinforce the irreplaceable value of empathy, touch, and relational care.
Equity and Access	Ethical foundation of ACPM	Ensuring inclusive digital transformation	ACPM supports leaders in identifying inequities in AI adoption by auditing disparities in resource allocation, student access to technology, and faculty readiness. Targeted support programs can help maintain fairness in learning outcomes and prevent the widening of digital divides.

### Limitations and recommendations

This study has several limitations. First, the findings reflect qualitative data from a specific institutional and cultural context in Türkiye; although participants were drawn from diverse universities and regions, caution is warranted in transferring the results to other countries or educational systems. Future mixed-methods and longitudinal studies could examine the validity and applicability of the ACPM across different nursing and midwifery programs and over time. Second, participants’ accounts primarily capture early-stage AI and robotics integration; given rapid technological change, perceptions, ethical concerns, and implementation strategies may evolve. Third, because data were collected via online FGDs, findings may be influenced by social desirability bias and the virtual format may have limited non-verbal interaction despite field notes. Finally, while NPT provided a useful interpretive lens, it may not fully capture ethical reasoning, emotional engagement, or socio-political dynamics; future research could incorporate socio-material or critical digital pedagogy approaches to offer a more comprehensive account of human-centred learning in AI- and robotics-enhanced nursing and midwifery education.

## Conclusion

This study examined how nursing and midwifery faculty members in Türkiye integrate artificial intelligence and robotics into education through the lens of NPT. Findings indicate that educators do not view AI as a simple technological add-on; rather, they described its potential to reshape teaching practices, professional roles, and approaches to person-centred care, while emphasizing that it should augment rather than replace empathy, presence, and relational care. Participants also highlighted that digital transformation is a collective, system-level process influenced by institutional readiness: disparities in infrastructure, leadership engagement, and policy guidance were reported as barriers, whereas collaborative culture and organizational ownership supported more sustainable integration in the Turkish higher education context. By mapping these accounts onto NPT constructs, we developed the ACPM as a theory-informed framework to guide ethical, human-centred, and sustainable AI/robotics integration in nursing and midwifery education. While some challenges may be context-specific, the core processes represented in the ACPM may be transferable to comparable educational settings with local adaptation. Future research should test the ACPM across diverse cultural and institutional contexts using mixed-methods and longitudinal designs, including intervention studies evaluating outcomes such as student empathy, clinical reasoning, ethical competency, and readiness for technology-enhanced care.

## Supplementary Information

Supplementary Material 1.

## Data Availability

The data supporting the findings of this study are available upon request from the corresponding author. The data are not publicly available due to privacy or ethical restrictions.
